# Gene expression bias between the subgenomes of allopolyploid hybrids is an emergent property of the kinetics of expression

**DOI:** 10.1371/journal.pcbi.1011803

**Published:** 2024-01-16

**Authors:** Hong An, J. Chris Pires, Gavin C. Conant

**Affiliations:** 1 MU Bioinformatics and Analytics Core, University of Missouri, Columbia, Missouri, United States of America; 2 Department of Soil and Crop Science, Colorado State University, Fort Collins, Colorado, United States of America; 3 Bioinformatics Research Center, North Carolina State University, Raleigh, North Carolina, United States of America; 4 Program in Genetics, North Carolina State University, Raleigh, North Carolina, United States of America; 5 Department of Biological Sciences, North Carolina State University, Raleigh, North Carolina, United States of America; University of Michigan, UNITED STATES

## Abstract

Hybridization coupled to polyploidy, or allopolyploidy, has dramatically shaped the evolution of flowering plants, teleost fishes, and other lineages. Studies of recently formed allopolyploid plants have shown that the two subgenomes that merged to form that new allopolyploid do not generally express their genes equally. Instead, one of the two subgenomes expresses its paralogs more highly on average. Meanwhile, older allopolyploidy events tend to show biases in duplicate losses, with one of the two subgenomes retaining more genes than the other. Since reduced expression is a pathway to duplicate loss, understanding the origins of expression biases may help explain the origins of biased losses. Because we expect gene expression levels to experience stabilizing selection, our conceptual frameworks for how allopolyploid organisms form tend to assume that the new allopolyploid will show balanced expression between its subgenomes. It is then necessary to invoke phenomena such as differences in the suppression of repetitive elements to explain the observed expression imbalances. Here we show that, even for phenotypically identical diploid progenitors, the inherent kinetics of gene expression give rise to biases between the expression levels of the progenitor genes in the hybrid. Some of these biases are expected to be gene-specific and not give rise to global differences in progenitor gene expression. However, particularly in the case of allopolyploids formed from progenitors with different genome sizes, global expression biases favoring one subgenome are expected immediately on formation. Hence, expression biases are arguably the expectation upon allopolyploid formation rather than a phenomenon needing explanation. In the future, a deeper understanding of the kinetics of allopolyploidy may allow us to better understand both biases in duplicate losses and hybrid vigor.

## Author summary

Allopolyploidy, or polyploidy through hybridization, is common across the eukaryotes. In newly formed allopolyploids, one of the contributing progenitor subgenomes generally expresses its genes at higher levels than does the subgenome from the other progenitor. It is believed that, as allopolyploids age, these expression differences drive differential duplicate gene losses between the subgenomes, as most older polyploidies show such biases in their gene losses. However, why the expression biases appear in the first place has been debated. Here, we use simple models of gene expression to show that the merging of distinct progenitor genomes through allopolyploidy will almost always yield expression biases due to the nonlinear kinetics of transcription. We argue that expression biases are hence the expectation for most allopolyploidies without a need for more complex explanations.

## Introduction

Hybridization held an odd place in evolutionary theory toward the middle of the last century, because geneticists’ view of its importance could vary substantially depending on their organism of interest [1]. In 1959, Stebbins [2] argued that zoologists working on terrestrial vertebrates tended to discount hybridization as those animals rarely formed fertile, reproductively isolated, hybrids. The reasons for this rarity probably center around these organisms’ obligate sexual reproduction and their common use of chromosomal sex determination. In contrast, he showed that there was clear evidence for the formation of new species through hybridization among the flowering plants [2].

Stebbins also discussed the differing routes by which such hybrids might form and laid particular emphasis on hybridization coupled to polyploidy, or allopolyploidy. It is now clear that flowering plant diversity has been hugely shaped by hybridization and in particular by allopolyploidy [3,4]. Genomic technologies have also provided evidence for many ancient hybridization and polyploidy events from across the eukaryotes (including vertebrates) that were not evident from morphological or cytological evidence alone [4–7]. These hybridizations are interesting for several reasons, not least because they can exhibit hybrid vigor or heterosis, meaning they possess desirable traits that exceed those of either of their progenitors [8,9]. Hybrid vigor is not usually explicable in terms of one or a few genetic loci, instead being driven by contributions from across the progenitors’ genomes [10]. One relatively simple explanation of this vigor would therefore be that the hybrid masks mildly deleterious homozygous recessive alleles in each progenitor lineage [10]. However, the differing heterotic behavior of different types of traits [11] and the differences seen in heterosis between polyploid and diploid hybrids [10,12,13] argue that other factors, termed overdominance, are also at work.

When comparing the different possible mechanisms of hybrid formation, hybridization through allopolyploidy presents a number of advantages: it does not require equal chromosome numbers to preserve fertility, it can produce essentially instantaneous reproductive isolation, and it can allow for the formation of hybrids between more distant lineages [2,3,14]. When coupled to the heterotic behavior of polyploids [10,12,13], these advantages of allopolyploidy may be part of the reason that allopolyploid plants were unusually likely to have been selected for domestication by early farmers [15].

As a reasonable number of recent allopolyploidy events are known, we can begin to explore and untangle the effects of polyploidy and hybridization by studying the functional genomics of these *neopolyploids*. One very important characteristic they often show is an unequal contribution to gene expression between the progenitor genomes. Commonly, one of the two progenitors shows higher average gene expression than the other in the allopolyploid [16–20]. Curiously, when we consider much older paleopolyploidies, there is also usually a strong statistical bias in the number of duplicate genes lost between the progenitor subgenomes, a pattern termed biased fractionation [21–26]. One can easily hypothesize that the early expression biases created conditions whereby gene losses were favored from the less expressed progenitor subgenome [16–20,27], making biased fractionation a consequence of biased expression.

What this hypothesis leaves unanswered, however, is the source of these expression biases [27]. Researchers have sought to divide the potential sources of such biases into the “parental legacy” and the effects of polyploid formation [28]. In this framework, a legacy of observable expression differences, either local or global, between the diploid progenitors might map to similar differences in the allopolyploid [29]. Alternatively, the formation of the polyploid offspring might, immediately or in time, give rise to expression differences between the parental subgenomes that did not exist in the diploid progenitors [28].

While attractive, this neat division of sources of bias has certain limitations. Somewhat trivially, we should probably conceptually divide parental differences into those due to the actions of selection and those attributable to genetic drift in expression [30,31]. Likewise, in natural systems, the actual parental lineages are rarely extant, adding further difficulties to the identification of the legacy [28]. A degree of ambiguity in terminology has also arisen, with the term “genome dominance” or “genomic dominance” having been employed both in the sense of a global bias in allopolyploid gene expression toward one progenitor subgenome [32] and in an alternative sense of the allopolyploid expression level being indistinguishable from one of the two progenitors (the “dominant” genome) [33]. We will thus avoid the term genomic dominance to prevent confusion. Instead, we will use “expression bias” to refer to greater relative expression from one homoeologous gene (i.e., paralog due to polyploidy) and “global expression bias” to refer to the case where one of the two subgenomes experiences expression bias in its favor (much) more often that the other.

A final concern with the parental legacy model is that we should ask whether we are considering *all* differences between the parental genomes to be part of the legacy or only phenotypically-evident ones. This distinction is key, because there are well-known examples of gene regulatory circuits that are phenotypically identical but genetically very distinct [34]. Under such circumstances, we probably lack the intuition of how a polyploidy event would affect relative expression levels.

The question of the source of the biases has also be approached empirically [35]. The most popular hypothesis currently is that the progenitor subgenome with the higher transposable element content experiences a repression of those elements mediated by the other subgenome, with the knock-on effect of repressing the expression of its nearby genes [20,36,37]. This explanation does not fit neatly into the parental legacy/novel feature dichotomy above because the effect is indeed only observed in the polyploid, but it results from significant genetic differences that have accumulated between the parental lineages.

Here we propose that computational models can be very enlightening on the question of allopolyploid expression biases because they allow us to control all of the complexities just mentioned that complicate analyses in real organisms [28,35]. We will therefore ask “How easy is it to generate allopolyploid genomes with expression biases from diploid progenitors that are phenotypically identical in their expression?”

This framing of the problem removes selected and neutral differences in parental expression levels from the analysis, allowing us to ask whether expression biases *require* such a parental legacy. We instead focus on more indirect changes between the parental genomes, such as changes in their size. Size is a very important parameter because cellular volume tends to scale with genome size [38], with polyploid organisms showing larger volumes than their diploid relatives. However, it is also important to recall that this scaling is not usually perfectly linear [39]. Our approach follows that Bottani and colleagues [19], who have pointed out that the kinetics of RNA polymerase binding and transcription will differ between genomes of different sizes, because, in a bigger genome, there are more sites for off-target polymerase (or transcription factor) binding. To achieve equivalent mRNA concentrations between a big and small genome, evolution will have needed to adjust the concentrations or the affinities of the transcriptional machinery in one or both genomes. An allopolyploid product of two such genomes would not, in general, show balanced expression between its two subgenomes.

Here we extend on this insight of Bottani et al. [19], showing that the null expectation of an allopolyploidy is unbalanced expression, particularly when the regulatory dynamics of the genes are relatively complex. Rather than a surprising result of allopolyploidy, we argue that subgenome-biased expression is the expected behavior of such hybrids.

## Results

### Modeling gene expression

Expression bias can be a global property of the genome. However, building an expression model of thousands of genes is computationally expensive and results in models that are difficult to interpret. Instead, we will first show how models of single genes respond to polyploidy and then discuss how some of the parameters of those models represent global quantities determined by the genome. The allopolyploid’s progenitor genomes will therefore be assumed to have had many generations to diverge in their transcriptional kinetics but will be required to have identical phenotypes. Throughout, we will use steady state mRNA concentrations in our measures of bias so that our results are independent of genome size and cell volume.

### Expression balance in hybrids is difficult to achieve and unstable

As an illustration of how a bias in expression could emerge immediately upon allopolyploid formation even with phenotypically identical parental lineages, we created models of an orthologous gene in two progenitor genomes A_1_ (ancestor 1) and A_2_ (ancestor 2). A_1_ and A_2_ differ in the DNA breathing rate [40–42]. This “breathing” is modeled with an opening (*k*_*o*_) and a closing (*k*_*c*_) rate parameter: the proportion of time that the DNA is transcriptionally active can be computed with the ratio of these two parameters. Their values will depend on a number of factors such as the base composition of the sequences in question [43].

**Fig 1:**
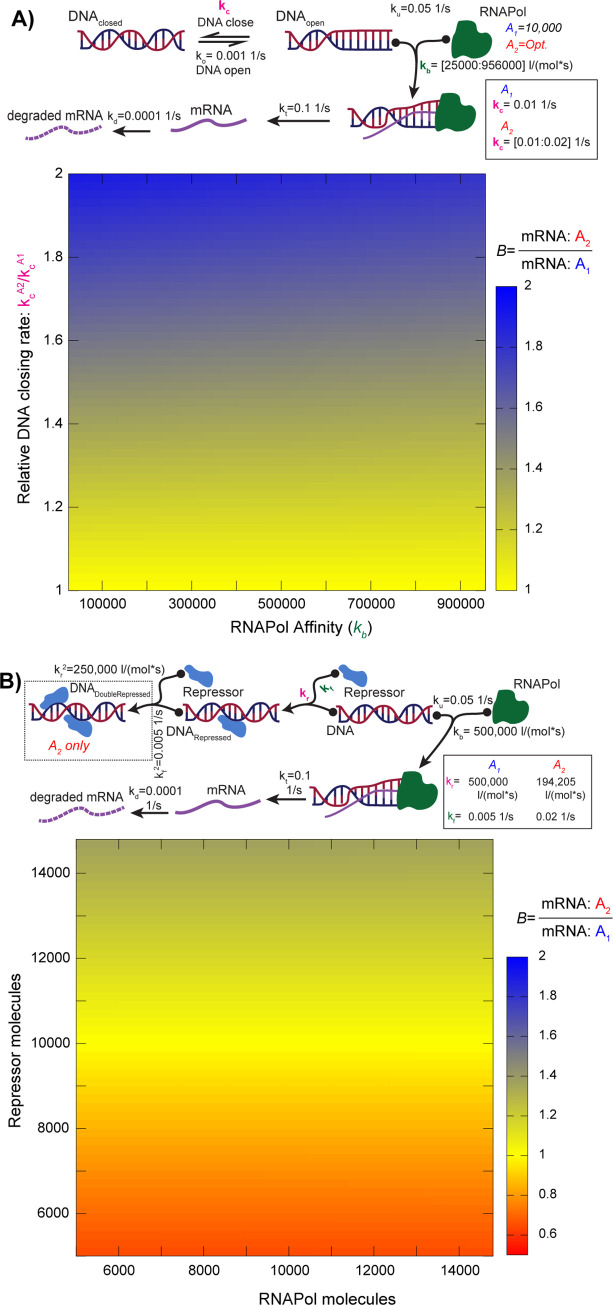
Two types of gene expression model that generate expression bias after allopolyploidy. **A.** In this model, the DNA transitions between a transcriptionally-available state (DNA_open_) and a closed state (DNA_closed_). The binding and unbinding of the polymerase to the open DNA then occurs at rates *k*_*b*_ and *k*_*u*_, respectively. Transcription is modeled as an irreversible process competing with polymerase unbinding (rate *k*_*t*_). The decay of the resulting mRNA then occurs on a timescale of minutes (rate *k*_*d*_). We model genes in two genomes A_1_ and A_2_, one of which has DNA that spends less time in the open configuration (*k*_*c*_*; y-*axis) and compensates with a higher effective concentration of RNA polymerase, such that the steady-state mRNA concentration is identical in A_1_ and A_2_. (The kinetics of transcription are also identical for the two). The allopolyploid hybrid of A_1_ and A_2_ has a doubled nuclear volume (2x10^-13^ l) and an RNA polymerase molecule count equal to the sum of those of A_1_ and A_2_. The heat map shows how *B*, the ratio of steady-state mRNA in A_2_ over A_1_, varies with RNA polymerase affinity (*k*_*b*_; *x-*axis) and the relative DNA closing rate in genome A_2_ (*k*_*c*_^*A2*^*/ k*_*c*_^*A1*^*; y-*axis). **B.** A more complex expression model, showing situations where balance after allopolyploidy can be achieved. Model A_1_ has a single repressor binding site which prevents transcription when a soluble repressor molecule is reversibly bound (rates *k*_*r*_ and *k*_*f*_ for binding and release, respectively). The second genome (A_2_) has two such sites that bind cooperatively: the second site has an increased binding rate *k*_*r*_^*2*^ and a reduced release rate *k*_*f*_^*2*^. The values of *k*_*r*_^*2*^ and *k*_*f*_^*2*^ are tuned, such that at a baseline level of repressor and RNA polymerase (10,000 molecules of each), the two cells have identical steady-state mRNA levels. Under these conditions, the allopolyploid hybrid P also has unbiased expression (*B =* 1.0). We show the value of *B* for a range of values of RNA polymerase (*x-*axis) and repressor (*y*-axis) levels from the allopolyploid hybrid.

In the two models we are comparing, progenitor A_2_ has its DNA transcriptionally active less often (higher *k*_*c*_; Fig 1A) than does A_1_. It compensates with higher RNA polymerase levels, such that A_1_ and A_2_ have identical steady-state mRNA concentrations. We created a model of an allopolyploid hybrid P by merging the two models, doubling the nuclear volume and assuming that P has an RNA polymerase concentration that is the arithmetic average of those of A_1_ and A_2_ (*Methods*). As one would expect, as the closing rate *k*_*c*_ from A_2_ increases, the bias against mRNAs from that subgenome increases (Fig 1A).

These models are an existence proof for instantaneous expression bias but are highly simplified and do not give a sense of whether bias is common. In Fig 1B, we use more complex models where the transcription levels result from competition between a repressor and the polymerase. In the model for species A_1_, the gene has only a single repression binding site, while A_2_ has two. We tuned the repressor affinities in A_2_ such that the DNA is exposed for transcription the same proportion of the time in A_1_ and A_2_. As a result, the two models have identical steady-state mRNA levels. When we form the allopolyploid hybrid, we find that no bias is seen across any concentration of RNA polymerase because of the equal DNA exposure. However, if we force the repressor concentration to change in P, as might happen if the volume of the allopolyploid did not experience perfect two-fold scaling [39], we see that bias once again appears. Hence, balance in mRNA levels is generally unstable, even in situations where the two progenitors are “tuned” to give it.

**Fig 2:**
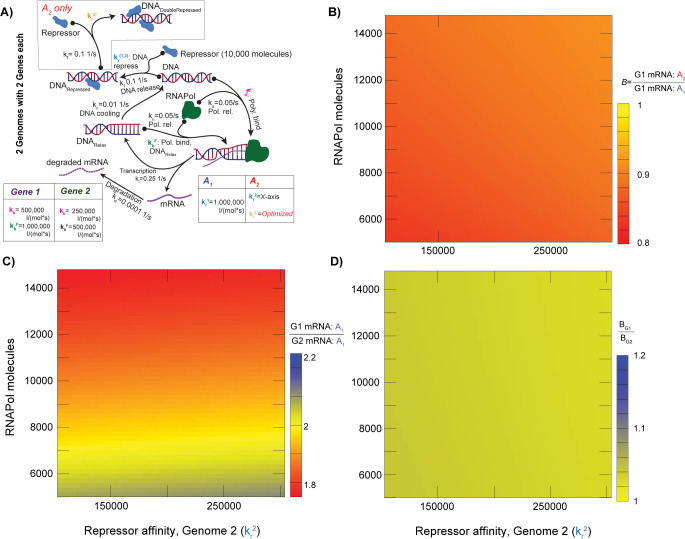
Gene expression differences and expression bias produced through allopolyploidy. **A.** The model is an extension of **Fig 1B**, such that transcription makes the DNA more accessible (DNA_Relax_), with a higher RNA polymerase affinity (*k*_*b*_^*F*^ verses *k*_*b*_). The relaxed DNA “cools” back to the DNA_open_ state at rate *k*_*c*_. Two genes G1 and G2 are modeled on separate DNA sections, with differing RNA polymerase affinities (*k*_*b*_), such that they differ by approximately a factor of 2 in their expression relative to each other. By optimizing *k*_*r*_^*2*^_,_ the steady-state mRNA levels for G1 and G2 are kept identical in A_1_ and A_2_ for all values of *k*_*r*_ in A_2_ (*x-*axis in **B-D**) and RNA polymerase level (*y*-axis). **B.** The bias *B* in G1’s mRNA between A_2_ and A_1_. **C.** Relative expression of G1 over G2 from subgenome A_1_ in P: A_1_ shows a roughly 2-fold difference in isolation. **D**. Shown are the (very slight) differences in bias *B* between G1 and G2 for the range of RNA polymerase and repressor affinities tested (A_2_/A_1_ for G1 over A_2_/A_1_ for G2).

Of course, genomes contain many genes, and it is important to understand how their relative expression levels and bias interact in hybrids. In Fig 2, we show a model that includes two genes, G1 and G2. At a per-gene level, this model is similar to that of Fig 1B except that we now make transcription autocatalytic, in the sense that DNA that has just been transcribed is more open to the binding of a new polymerase molecule [44]. We tuned the affinity of A_2_’s second repressor site so as to make the steady-state mRNA levels of A_1_ and A_2_ identical for any combination of RNA polymerase concentration and affinity of A_2_’s first repressor site (*Methods*). Unsurprisingly, gene G1 in the allopolyploid hybrid shows expression bias, a bias that varies as polymerase levels and repressor affinities change (Fig 2B). Strikingly, the bias observed for G2 is very similar to that for G1 (Fig 2D). If, in real organisms, one observed such similar bias levels between pairs of genes, one would be tempted to infer that the expression ratio between G1 and G2 in the allopolyploid was reflective of its ratio in the progenitors. However, such is not the case: Fig 2C shows that the G1/G2 expression ratio from subgenome A_1_ in the allopolyploid varies considerably across the range of polymerase concentrations, even though it is constant at effectively 2:1 in the ancestral A_1_ genome. In other words, knowing that two pairs of homoeologous genes have similar biases in an allopolyploid does not allow us to conclude that the relative expression level that we see for those two pairs of genes in that allopolyploid reflects their relative expression levels in the progenitor lineages. This limitation holds despite the fact that the two progenitors have identical expression levels for *both* genes.

### Most pairs of expression models with identical expression produce bias when hybridized

**Fig 3:**
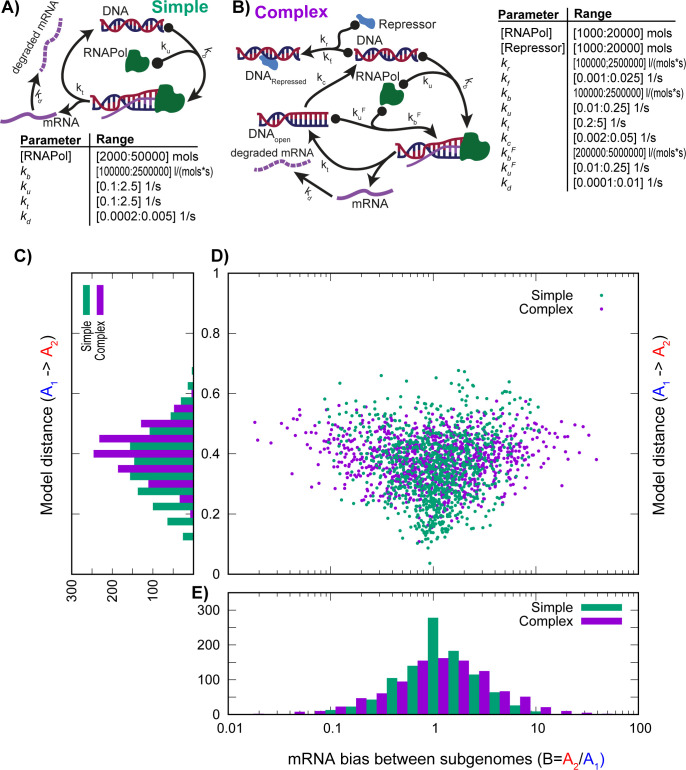
Exploring the state space of expression models for allopolyploids. In **A&B**, we propose two meta-models of gene expression, a simple one (**A**) involving only the polymerase binding a target gene (similar to Fig 1A but without DNA breathing), and a more complex one that follows **Fig 2** with slightly different parameter values (*Methods*). In each case, we generate two random models A_1_ and A_2_ by selecting uniform random values for the model parameters across the ranges listed (*Methods*). After computing the steady state mRNA concentration for A_1_, we use an approximate gradient method to bring A_2_ to equal steady state mRNA concentration with A_1_ (*Methods*). The resulting parameter values are normalized and the Euclidian distance between A_1_ and A_2_ is computed (*Methods*). We then construct an allopolyploid hybrid of A_1_ and A_2_ and compute resulting mRNA bias *B* **C.** Distribution of model-pair distances for 1000 simulations of the simple and complex models from **A&B**. On *x* is the distance between the model pairs (*y-*axis in **D**), on *y* are the frequencies of those distances. **D.** One thousand random pairs of genome models were created from each meta-model and their hybrids simulated. The plot shows the relationship between the distance between the model pairs (*y*-axis) and expression bias *B* (*x*-axis: note the log scale). **E.** Histograms of *B*, plotted on a log-scale (*c*.*f*., **C**).

The above results show that allopolyploids need not necessarily show balanced expression between their subgenomes. But of course, it is possible that we have, by chance or design, selected model parameters that give the misleading impression that bias is common. What if instead, as is seen in metabolic pathways [45], expression in allopolyploids is canalized, such that most expression configurations do not produce imbalances? To address this concern, we assessed the prevalence of bias for pairs of models that randomly sampled the parameter space. To do so, we defined a range of generally sensible parameter values and uniformly and randomly sampled from them to define models of genomes A_1_ and of A_2_. Of course, such model pairs will essentially never have equal mRNA levels. So, for model A_2_, we used step-wise optimization to bring the mRNA concentrations to equality with those from A_1_ (*Methods*). Doing so does not make the models of genomes A_1_ and A_2_ identical: the parameter values for A_1_ and A_2_ are generally dissimilar (Fig 3). Across 1000 pairs of random models, expression biases, even very large ones, are the rule rather than the exception: only 16% of the pairs from the more complex metamodel of Fig 3B have expression biases less than ±1.25 fold. There are three conclusions we can draw from this analysis. The first is that, even when the underlying expression meta-model is structurally identical, there is an enormous range of potential parameter values that can give equal expression levels. However, the second conclusion is that the formation of an allopolyploid from those models almost invariably results in expression bias. Our final conclusion is that canalization does not seem to be at play, because using a more complex expression meta-model produces more, not less, bias (Fig 3D).

**Fig 4:**
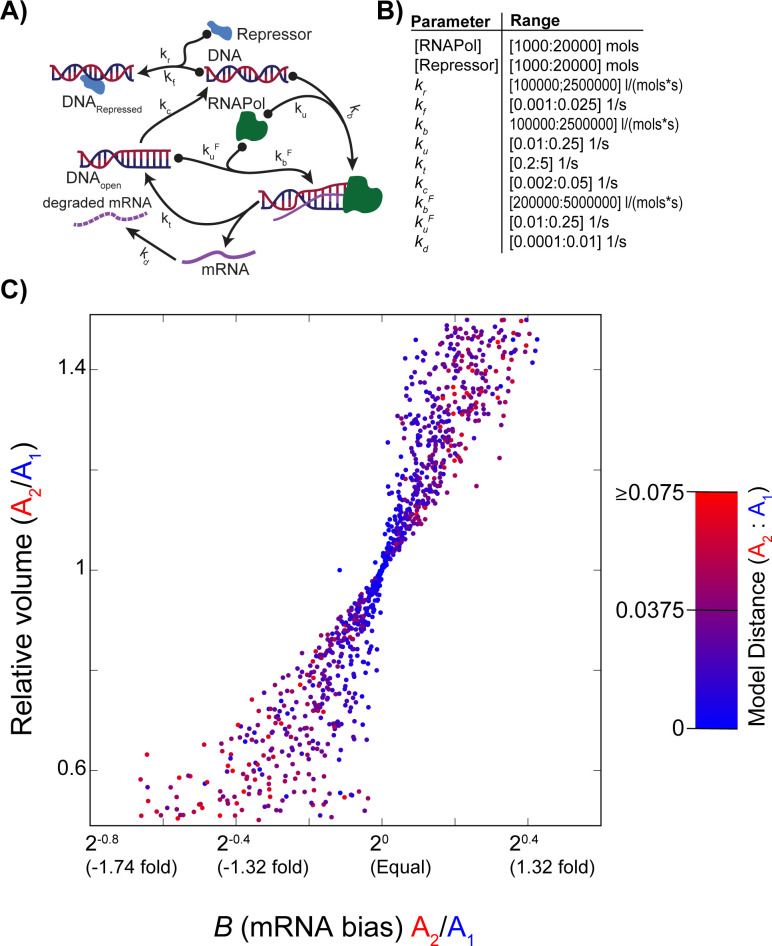
Hybrids formed from genomes of differing sizes show expression bias. **A)** Meta-model of gene expression used. Random example models were created from this model by drawing uniform random variables for each parameter across the range shown in **B**. From these random models, we created two progenitor genomes (A_1_ and A_2_) with initially identical kinetics, but where A_2_ could have a 50% smaller or larger genome (uniform random variable on [0.5,1.5]; *y*-axis; see *Methods*). We adjusted A_2_ to give identical steady-state mRNA levels to A_1_ and then formed the hybrid to have a volume equal to the sum of the volumes of A_1_ and A_2_ (*Methods*)_._ **B)** Table of parameter ranges. **C)** Bias in steady-state mRNA levels seen for 1000 different random expression models. On the *x*-axis is the bias *B* and on *y* is the ratio of the volume of A_2_ to A_1_. Points are color-coded by the Euclidian distance in normalized parameter values between A_1_ and A_2_ (*Methods*): the maximum distance observed was 0.14.

Fig 3 makes the unrealistic assumption of a hybridization between two effectively unrelated progenitors. Can closely related progenitors also display bias after hybridization? To address this question, we created pairs of models A_1_ and A_2_ where A_1_ was created at random in the manner just described, but where A_2_ was simulated to have a genome size between 50% smaller and 50% larger than A_1_. Genome size was modeled as a change in nuclear volume, given the strong association of these two values [38,46]. The A_2_ models were initialized with kinetics identical to A_1_, but then adjusted as just described to give equal steady-state mRNA levels despite their differing volumes (*Methods*). Even pairs of models with rather small differences in their parameters can give expression biases in the face of genome size differences (Fig 4). In fact, only 52% of the simulations had expression levels within 1.1 fold of each other.

### From genes to genomes

The models we have described of course only consider individual genes. Directly applying an approach such as that used in Figs 3 and 4 across an entire allopolyploid genome would produce a distribution of expression biases between A_1_ and A_2_ with a large variance. However, because the model parameters from each gene would be independent, the *mean* bias for that allopolyploid would be zero. In other words, no global expression bias toward one subgenome would be observed. But are there conditions where the effects we have modeled could give rise to a global expression bias?

**Fig 5:**
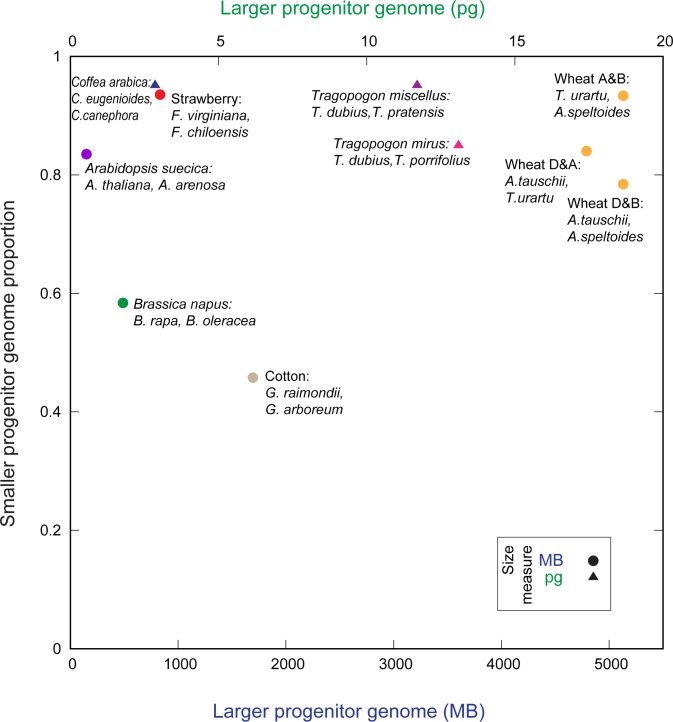
Recent allopolyploidies have formed from progenitors with genomes of different sizes. Eight recent allopolyploidies are shown for which the progenitor genomes are known. On *x* is the size of the larger of the progenitors in megabases of DNA (MB, lower axis) or in picograms of DNA per 4C (upper axis). On *y* is given the proportional size of the smaller progenitor subgenome. Because bread wheat is an allohexaploid, the three possible comparisons of the three progenitors are shown individually. Data sources for the genomes shown are given in the S1 Table.

A natural place to start looking for such patterns would be in the *sizes* of those progenitor genomes. Fig 5 gives the genome sizes of the progenitors for several recent allopolyploidy events in flowering plants where those progenitors are known with some confidence [32,47–52]. Although in a few cases there is less than a 10% difference in size between the progenitors’ genomes, in only *T*. *miscellus* and *C*. *arabica* are the differences less than 5% (4.9% in both cases).

As is suggested by Fig 4, a simple change in genome size necessarily requires changes in global transcriptional regulatory dynamics in order to maintain gene expression patterns. Bottani et al. [19] have already described this issue, pointing out that, in a larger genome, transcription factors experience more off-target binding, reducing their occupancy of the true transcription start sites. The important question is how a genome would compensate for its size increasing over evolutionary time. The intuitive answer, given by Bottani et al. [19], would be to increase the affinities of the transcription factors and their binding sites. However, individually tuning all these local affinities across all the transcription factors and binding sites in the genome would be a slow process. A more rapid adaptation would be to globally repress non-genic DNA. In the models above such repression might be accomplished by a higher DNA closing rate (Fig 1A) or greater numbers of repressor binding sites (Fig 1B). Mechanistically, such repression could involve a combination of DNA methylation, histone modifications and changes to the three-dimensional organization of the chromosomes in the nucleus [53–55]. In fact, such changes might even be automatic: mammalian cells can respond to differences in their volumes relative to a constant DNA content by changing their burst transcription dynamics [56], suggesting the existence of some feedback between cell volume, DNA content and the transcription process. In all of these cases, the result would most likely be a pair of genomes that were phenotypically identical but that would form allopolyploid hybrids possessing global expression biases (c.f., Fig 1B, where balanced expression is only seen with very precise parameter tuning).

Looking beyond even genome size, we can notice that our models in fact include both local and global controls on gene expression. Parameters such as the RNA polymerase binding constants (*k*_*b*_ and *k*_*u*_) are primarily local: that is to say specific to a gene and its promotor. On the other hand, changes in DNA opening and closing, as well as repressor affinities and (especially) repressor and polymerase concentrations, are more likely to be global genomic responses. In other words, they would be expected to be the factors evolution might adjust in response to changes in genome size or base composition (see below). Allopolyploids formed from progenitors that differed in such factors would tend to have global expression biases favoring one progenitor.

### Extant allopolyploid genomes have many features that could drive hybridization biases

**Fig 6:**
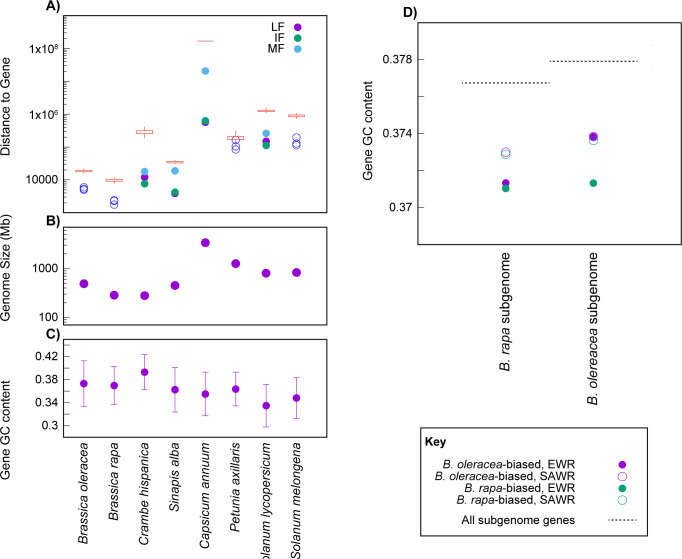
Mesohexaploid genomes vary several aspects of genome structure in ways relevant to the formation of further hybrids. **A)** We identified tRNA genes in the intergenic region of each of eight genomes with shared mesohexaploidies and then computed the mean distance of those genes to the nearest protein coding gene for which we could identify the subgenome of origin (*y*-axis; see *Methods*). Those subgenomes vary in their level of gene preservation from most surviving genes (least fractionated, LF), through intermediate and most fractionated (IF and MF). We compared these distances to randomly distributed tRNAs, finding that in all cases, the tRNAs were closer to surviving genes than expected (*P* = 0.02 for *P*. *axillaris* and P<0.01 for all other genomes; note the log-scale on *y*). In four cases, the subgenomes differed from each other in their mean distance to the tRNA genes more than would be expected (colored points). Genomes also differ in total size (**B**) and in the average GC content in the genes (mean ±2 standard deviations; **C**). **D)** Finally, both European and South Asian winter rapeseed accessions of *B. napus* (EWR and SAWR, respectively) show lower average gene GC content for genes with highly biased expression between paralogous pairs compared to most paralogous gene pairs (*Methods*).

How applicable are these theoretical findings to real allopolyploids? While genome size is a useful conceptual framework for thinking about the problem, it is only one of many factors driving a genome’s transcriptional dynamics. Differences in transposable element load have already been discussed as a potential source of expression biases [20,36,37]. Unfortunately, for older allopolyploidy events, this hypothesis is difficult to test because of the rapid evolutionary turn-over of these elements [57]. In some recent analyses, we found a tendency for tRNA genes to be overly frequent in some of the subgenomes of the hexaploid Brassicas, but no similarly strong trend in the hexaploid Solanaceae [57]. That pattern would be consistent with the ideas presented here; the expression and loss biases in any given allopolyploid will be due to the combination of many components of genome structure, including repetitive elements, genome size, the dynamics of genome repression, and GC content, among others. In Fig 6A–6C we show the range of variation in these factors across eight paleohexaploid genomes. We consider genome size for the reasons discussed above, tRNA distance due to our observations in the *Brassicas* [57] and GC content because DNA melting and hence opening and closing should differ between regions with differing base composition (c.f., Fig 1A). A potentially intriguing observation is that, in some cases, the three subgenomes produced by ancient hexaploidies differ amongst themselves in the average distance between their genes and tRNA loci (Fig 6A), consistent with the idea that features like the local transcriptional environment can affect a gene’s survival propensity.

We also see that such factors can measurably alter the expression bias seen after polyploidy. *Brassica napus* is an allopolyploid hybrid of *Brassica rapa* and *Brassica oleracea* and shows significant expression bias toward the *B*. *oleracea-*derived subgenome (*Methods*). The fact that the bias, while significant, is relatively modest is probably due to a history of subgenome replacements by homoeologous exchange in this plant [52].

When we look at the 200 most extremely *B*. *rapa-*biased homoeologous gene pairs or the 200 most *B*. *oleracea*-biased pairs, these genes have significantly lower gene-wide GC content for both homoeologs than do other genes (*P≤*0.018, randomization test, Fig 6D). Likewise, the two subgenomes differ in their gene distributions, with the *B*. *rapa-*derived subgenome having more closely spaced genes, consistent with the smaller *B*. *rapa* genome (*P* = 0.001, randomization test). It appears that this difference in gene spacing may also affect expression in the two subgenomes: there is a stronger correlation of distance to the nearest gene and expression similarity for the *B*. *rapa-*derived subgenome than for the *B*. *oleracea*-derived one (see *Methods*).

Strikingly, a recent experiment on *B*. *napus* showed that at least some of the expression bias in these subgenomes is attributable to differences in their chromatin accessibility, with ATAC-Seq showing the *B*. *oleracea* subgenome to have more accessible chromatin regions than the *B*. *rapa* subgenome [58]. While the reasons for these chromatin structure differences between subgenomes are not clear, they represent just the type of genomic differences that would be expected to yield subgenome bias after hybridization.

## Discussion

After a pair of populations cease to interbreed, they can diverge in a variety of ways. Here we have made the simplifying assumption of only considering divergence that does not change the phenotype. In effect, we are constraining evolution to occur along a *neutral network* of genotypes of equivalent phenotype [59]. This assumption is probably less restrictive than it appears. If one categorizes the genomic changes affecting gene expression as being due either to *cis* (local) or *trans* (elsewhere in the genome) effects [60], it is reasonably common to observe *compensatory* changes, where a *trans* change in one direction is accompanied by a *cis* change in the other [61]. A natural explanation for these compensatory changes is stabilizing selection to maintain gene expression levels over time. If so, our assumption of identical expression levels is probably a reasonable one at the genome-wide level, even though of course some individual genes will deviate from it [60].

We asked whether expression biases are still common under this assumption, and what kind of transcriptional responses to polyploidy are generally seen. It is very clear that neutral changes in transcription dynamics will drive expression biases at the level of individual genes. It is likely, though less certain, that these types of changes will also produce global expression biases. For instance, in the case of a genome size change, the dilution effects of the larger genome could be compensated for by either increasing the promotor affinities of all genes or by a higher expression of the RNA polymerase [19]. The latter change is probably more evolutionarily accessible because it requires fewer individual mutations to achieve. Under that mechanism, *trans* changes would have accumulated as genome size increased, with later *cis* changes fine-tuning the expression of individual genes. That sequence is potentially compatible with the general observation that *cis* expression changes seem to accumulate over evolutionary time without producing correspondingly increasing levels of expression changes [61].

While in the prior example the larger genome is in some sense in the weaker position, we should not assume that this is always the case. For instance, as seen in *B*. *napus*, isolating genes within large regions of heterochromatin could reduce coupling in their expression levels [62], allowing more precise expression control of each. In this scheme, a physically larger genome might show both fewer cases of pairwise correlation in expression between neighboring genes and less off-target binding, if most of that excess DNA were kept in a heterochromatic state. For researchers, the downside to all of this complexity is that it will be hard to predict *a priori* how a hybrid of two genomes will behave with respect to expression dominance.

If the prediction of the favored subgenome remains elusive, our models strongly suggest that that such genomic differences, resulting as they do from nonlinear kinetic differences between the lineages, mean that allopolyploid hybrids are unlikely to show globally balanced gene expression. Thus, if bias is the rule for the quite simple models considered here, it seems unreasonable to believe that real genomes, with potentially thousands of different types of molecules contributing to expression levels, could commonly produce balanced expression, particularly because allopolyploids are not generally thought to be perfect “two-fold” copies of their progenitors [39]. Among the factors that might affect global biases are changes in DNA methylation: in neopolyploid *Mimulus* plants, methylation patterns are disrupted at polyploid formation and over time reestablish themselves in a manner favoring the dominant subgenome [16]. Likewise, the nature of the regulatory circuits involved is likely important: genes with putative dosage interactions tend to show instantaneous responses to allopolyploidy that are more similar to each other than expected by chance [63]. Therefore, we suggest that the hypothesis of global expression biases being driven by differences in transposon load [20,36,37] can be complemented by the more general patterns seen here. One difference in the two positions is that the phenomena considered here do not make the assumption that factors in one progenitor subgenome must act in a specific way on the other.

Our models do not speak directly to the question of the later biases in duplicate losses, although, as mentioned, global expression biases are expected to contribute to global loss biases. Nothing in these results gives us estimates of what level of global expression bias is needed to result in a duplicate loss bias. However, we should at least recall that such biases need not be as large as the expression differences that we would require to, say, describe a pair of genes as differentially expressed in a direct comparison [64]. Hence, if expression biases are the rule after allopolyploidy, duplicate losses biases are probably expected to follow on later.

We cannot yet draw any firm conclusions on questions regarding heterosis and hybrid vigor either. However, we do believe that a modeling approach creates a framework for thinking about the problem, reinforcing the message that implicit linear models in evolution can be misleading. Hybrids are not generally expected to be the average of their parents for complex biochemical features such as gene expression. As a simple illustration, consider an aspect of expression we have disregarded: expression noise [41,65]. The formation of an allopolyploid, by doubling the number of genes, should have the side effect of reduced noise in gene expression [42,65,66]. Hence, we might ask whether one source of heterosis in alloployploids is greater predictability in their gene expression patterns. Perhaps a more general version of this insight is possible, with heterosis being explicable in light of the complex interactions between the genomes and the mechanics of how their genes are expressed: testing such ideas will require a much more granular sense of those mechanics and their genetic control.

## Methods

### Overview of models of gene expression

We hybridized models of gene expression for two distinct species A_1_ and A_2_. The hybridization creates a new allopolyploid cell P with all four homoeologous gene copies present in a single nucleus of doubled volume. For simplicity, the progenitors were each assumed to have a nuclear volume of 10^−13^ l, [1/5 of the volume of a human nucleus; 67]. The steady-state mRNA levels A_1_, A_2_ and P were computed with COPASI 4.36 [68]. Because A_1_ and A_2_ have identical volume and P has double that volume, the mRNA particle numbers computed by COPASI can be treated as concentrations in our analyses. For P we computed the expression bias *B*: namely A_2_’s steady-state mRNA level divided by that of A_1_. We considered several types of expression models to better understand the behavior of *B*.

### Chromatin relaxation model

This model considers the transition from closed, non-transcribable chromatin to open, accessible, chromatin [69,70] to be the rate-limiting factor in transcription. It is consistent with data measuring noise in mRNA levels [40,41,71,72]. Following Suter et al., [73], we model the transition between closed and open chromatin as occurring on a timescale of tens of minutes (Fig 1A), with the mRNA half-life being ten-fold longer. A_1_ and A_2_ differ in the proportion of the time that their DNA remains open (*k*_*c*_*/k*_*o*_). The reversible binding of the polymerase to the promotor was modeled as showing roughly a ten-fold lower binding rate (*k*_*b*_) than seen in bacteriophages [74] but with also a stronger affinity for the polymerase (a *k*_*u*_ of 20-fold lower; Fig 1A), corresponding to need for higher promotor affinity in larger eukaryotic genomes. Transcription was modeled as an irreversible process occurring on a timescale of seconds (*k*_*t*_; Fig 1A). We explored how the mRNA bias in the polyploid varied with the affinity of the RNA polymerase (*k*_*b*_) for the gene’s promotor. We fixed the number of RNA polymerase II molecules in model A_1_ at 10,000 copies, in rough accordance with data for the RPB2 subunit from yeast [75]. For each combination of polymerase affinity *k*_*b*_ and *k*_*c*_*/k*_*o*_ in A_2_, we optimized the concentration of RNA polymerase to give the same mRNA levels as seen in A_1_ for that value of *k*_*b*_. Steady-state mRNA levels for A_1_ and A_2_ ranged between 0.5 and 19 molecules per cell. The polyploid offspring P was assumed to have the sum of the number of RNA polymerase molecules as did A_1_ and A_2_.

### Repression model

In this model, DNA is or is not available for transcription based on the binding of repressive factors (Fig 1B) analogous to repressive histone marks or DNA methylation. The kinetics of the RNA polymerase and transcription were kept the same as in the previous model. For model A_1_ we assumed that the repressor had similar binding kinetics as did RNA polymerase (*k*_*r*_ *= k*_*b*_ = 500,000 1/mol•s), but with a lower off-rate (*k*_*f*_ *=* 0.005 1/s verses *k*_*u*_ *=* 0.05 1/s Fig 1B). For model A_2_, we added a second repressor binding site that operates cooperatively with the first. Hence, if one of the two repressor sites is occupied, the binding of a repressor to the second occurs at a higher rate (*k*_*r*_^*2*^*>k*_*r*_) and its release at a lower rate (*k*_*f*_^*2*^*<k*_*f*_). We selected values for these four parameters (*k*_*r*_^*2*^, *k*_*r*_, *k*_*f*_^*2*^ *and k*_*f*_; Fig 1B) such that the proportion of time that the DNA spent in the unrepressed state was the same for A_1_ and A_2_. Since A_1_ and A_2_ also have identical transcription kinetics, they have identical mRNA concentrations. We explored the dependance of *B* on the allopolyploid’s number of repressor and RNA polymerase molecules. Steady-state mRNA levels for A_1_ and A_2_ ranged between 21 and 162 molecules per cell.

### Expression comparison model

We created a model with two genes G1 and G2 differing in their expression. Model A_2_ again had two repressor binding sites to A_1_’s single site. We added a relaxed state to the DNA model, corresponding to a gene that has just experienced transcription and has an enhanced affinity for the RNA polymerase (*k*_*b*_^*F*^*>k*_*b*_; Fig 2A). The DNA in this relaxed state returns to the normal open state over a time frame of minutes (*k*_*c*_) unless a second transcription event returns it to the relaxed state. Because this effect is included, the polymerase exits the promotor slightly more quickly than for the prior model (*k*_*t*_ = 0.25 1/s verses 0.1 1/s).

To explore the relationship between bias and relative expression level, G2 has an RNA polymerase affinity of half that of G1 (*k*_*b*_; Fig 2A). We explored the dependance of *B* on both the affinity of the repressor for A_2_’s first repressor binding and on the number of RNA polymerase molecules (Fig 2). For each such pair of values, the relative repressor affinity for A_2_’s second site (*k*_*r*_^*2*^) was optimized so as to give equal gene expression for both G1 and G2 between A_1_ and A_2_. In addition to the bias *B*, we also computed the ratio of the expression of G1 and G2 from A_1_ in the allopolyploid and the value of *B* for G1 over than for G2.

### Random models

We explored the bias across a range of random transcriptional models drawn from two meta-models: a simple one *S* and a complex one, *C*. Model *S* considers only transcription itself, with 4 parameters, *k*_*b*_, *k*_*u*_, *k*_*t*_ and *k*_*d*_, as well as an RNA polymerase concentration. We used central values for these parameters of *k*_*b*_ = 500,000 1/mol•s, *k*_*u*_ = 0.5 1/s, *k*_*t*_ = 0.5 1/s and *k*_*d*_ = 0.001 1/s, with a central polymerase molecule number of 10,000. These values of *k*_*t*_ and *k*_*d*_ differ slightly from the prior models to give a better sampling of parameter space. Hence, the *C* model was constructed with parameters similar to those of the A_1_ model of Fig 2 with adjustments to avoid too many models with invalid mRNA levels. The central value of *k*_*f*_ was reduced by a factor of 2, that of *k*_*t*_ increased from 0.25 to 1 and that of *k*_*d*_ raised from 0.0001 to 0.0005 (In all cases, the parameter values used in Fig 2 are within the range of the sampling for the random models used here).

We constructed pairs of random models for A_1_ and A_2_ as follows. For each parameter, we allowed a range of values from 5-fold under to 5-fold over the central value. To create A_1_, for each parameter we drew a uniform random number on its parameter interval. Using these parameters, we computed the steady-state mRNA level for the resulting random A_1_ model. If that level was less than 1 or greater than 300, the model was rejected. Otherwise, we retained that mRNA level and generated a random A_2_ model in the same way. We then computed an approximate derivative for each parameter in A_2_ and used those derivatives to match the mRNA level of model A_2_ with that of A_1_. Briefly, we increased each parameter in A_2_ by 10%, recomputed the mRNA level and calculated the slope between the parameter change and mRNA change. We then used that slope to adjust the parameter in the direction of the desired mRNA level from A_1_. We next recomputed the mRNA level for A_2_, as well as the changed parameter’s slope. If the two models still differed in their mRNA levels by 0.005 molecules or more, the next parameter was selected and the optimization continued. Once optimization was complete, if the optimized A_2_ model had parameters outside of the valid parameter ranges, that pair of models was discarded and a new A_1_ model selected.

Once a pair of models A_1_ and A_2_ had identical mRNA levels, we formed the polyploid model P, taking the sum of the RNA polymerase and repressor molecules for the two models and doubling the nuclear volume. We then computed *B* from P. Finally, we normalized the parameter vectors for A_1_ and A_2_ to the interval [–1,1] using the boundaries above and computed the Euclidian distance between A_1_ and A_2._

### Models with differing genome sizes

Using a similar approach, we also compared pairs of related progenitor models differing in genome size. To do so, we first proposed a random model A_1_ as just described. We represented the difference in genome size as a nuclear volume change [38,46] in the A_2_ model by drawing a nuclear volume on the random interval [5x10^14^,1.5x10^-13^] (±50% relative to A_1_). We set the model parameters of A_2_ to initially be identical to A_1_. We then computed A_1_’s steady-state mRNA level and used the gradient approach above to bring A_2_’s mRNA level to the same value. Notice that in this case only we sought equal mRNA molecule counts between models A_1_ and A_2_ rather than equal concentrations. We did so in order avoid creating artificial parental differences when the P model was formed. We then created P as before, making its nuclear volume the sum of those of A_1_ and A_2_ and computing the resulting value of *B*.

### *B*. *napus* gene expression data

Gene expression data from the allopolyploid *Brassica napus* were taken from our previous work [76]. We identified the pairs of homologous genes in the A and C subgenomes of *B*. *napus* (v 4.1) and *B*. *rapa* and *B*. *oleracea* [57], respectively, using GenomeHistory 2.0 [77]. We then used our previously described tool for orthology inference [78] to identify 1:1 orthologs between those subgenomes and the respective *Brassica* genome. Using POInT, our tool for identifying orthologous genes produced by polyploidy, we filtered these sets of orthologs to those where a paralogous pair in *B*. *napus* could be directly linked to a pair of high (≥95%) confidence orthologous genes from *B*. *rapa* and *B*. *oleracea* [79]. The result was 4858 paralogous *B*. *napus* genes with expression measurements for the paralogs from both the *B*. *rapa* and *B*. *oleracea* subgenomes. As expected from the arguments above, these subgenomes show a statistically significant expression bias toward the *B*. *oleracea* subgenome for both European and South Asian winter rape seed (EWR and SAWR, respectively, *P≤*0.003, randomization test).

For each of these genes, we calculated the local GC content extending 1000bp upstream and downstream of the annotated gene coordinates, as well as using BLASTN [80] and a tRNA database [81] to find the distance from each gene to its closest tRNA. We selected the 200 (4%) paralogous pairs most biased toward the *B*. *rapa* subgenome and the 200 most biased toward the *B*. *oleracea* subgenome and compared their average GC content to the average of all genes using a randomization test. Results are generally similar for using the top 100 and 500 most biased genes, but with 2 out of 8 comparisons being non-significant in each case (*P>*0.05).

We also examined the potential role of gene-to-gene distances in driving expression patterns across the *B*. *napus* subgenomes. For each subgenome, we compared the distance to the nearest gene with the relative expression difference between that pair of genes (difference in RPKM over mean RPKM for the pair). For the *B*. *rapa* subgenome, there is a weakly significant association between these two factors (more distant genes are less similar in expression, Pearson’s *r =* 0.058, *P* = 0.032 and *r* = 0.059, *P* = 0.028, for EWR and SAWR, respectively). (The values for the Spearman correlation are rho = 0.043, *P =* 0.11 and rho = 0.045, *P =* 0.09 for EWR and SAWR, respectively). However, this association is weaker and non-significant for the *B*. *oleracea* subgenome (Pearson’s *r =* 0.002, *P* = 0.94, *r =* 0.007, *P* = 0.79 for EWR and SAWR, respectively; Spearman’s rho = -0.029, *P* = 0.29, rho = -0.028, *P* = 0.31 for EWR and SAWR, respectively). When we compare these real associations to those seen when the subgenome identities are randomized, the difference in these associations between the two subgenomes is significantly larger than would be expected by chance for the Spearman’s rho (*P =* 0.03 and 0.029 for EWR and SAWR, respectively), though not for Pearson’s *r* (*P =* 0.068 and 0.088 for EWR and SAWR, respectively).

## Supporting information

S1 Table The supplemental table gives genome sizes and citations for the data shown in Fig 5. (PDF)
