# Supplementary material for: Gene expression bias between the subgenomes of allopolyploid hybrids is an emergent property of the kinetics of expression
Source: PLoS Comput Biol. 2024 Jan 16;20(1):e1011803. doi: 10.1371/journal.pcbi.1011803 (PMC10817154; doi:10.1371/journal.pcbi.1011803)
Supplement: S1 Table — (PDF) [file pcbi.1011803.s001.pdf]

Supplemental Table: Genome sizes for progenitors of recent allopolyploids

Progenitor Species	Allopolyploid descendent	Genome Size	Reference
Gossypium raimondii	Cotton (*G. hirsutum)*	775.2 MB	[1]
Gossypium arboreum	Cotton (*G. hirsutum)*	1,694 MB	[2]
Aegilops tauschii	Wheat (*T. aestivum*)	4,025 MB	[3]
Triticum urartu	Wheat (*T. aestivum*)	4,790	[4]
Aegilops speltoides	Wheat (*T. aestivum*)	5130MB	[5]
Fragaria chiloensis	Strawberry (*F.* × *ananassa*)	824.2-839.9MB[a]	[6]
Fragaria virginiana	Strawberry (*F.* × *ananassa*)	769.2-787.8MB[a]	[6]
Tragopogon dubius	*T. miscellus/ T. mirus*	10.8,10.83,11.76 pg[b]	[7]
Tragopogon pratensis	*T. miscellus*	11.08,11.59,12.44 pg[b]	[7]
Tragopogon porrifolius	*T. mirus*	13.7,12.50 pg[b]	[7]
Coffea eugenioides	*C. arabica*	1.36 pg[c]	[8]
Coffea canephora	*C. arabica*	1.43 pg[c]	[8]
Brassica oleracea	*B. napus*	488MB	[9]
Brassica rapa	*B. napus*	285MB	[10]
Arabidopsis thaliana	*A. suecica*	125MB	[11, 12]
Arabidopsis arenosa	*A. suecica*	149.7MB	NCBI: GCA_905216605.1[d]

[a]: Genome size used in Figure 5 is the average of these values.
[b]: Values given in picograms of DNA per 4C: points in Figure 5 are the average of the values given.
[c]: Values given in picograms of DNA per 2C: points in Figure 5 given at twice this value for comparison to *T. miscelluis* and *T. mirus*.
[d]: Genome size taken from the deposited reference sequence at NCBI.

References:

1. Wang K, Wang Z, Li F, Ye W, Wang J, Song G, et al. The draft genome of a diploid cotton Gossypium raimondii. Nature genetics. 2012;44(10):1098-103.

2. Li F, Fan G, Wang K, Sun F, Yuan Y, Song G, et al. Genome sequence of the cultivated cotton Gossypium arboreum. Nature genetics. 2014;46(6):567-72.

3. Luo M-C, Gu YQ, Puiu D, Wang H, Twardziok SO, Deal KR, et al. Genome sequence of the progenitor of the wheat D genome Aegilops tauschii. Nature. 2017;551(7681):498-502.

4. Ling H-Q, Ma B, Shi X, Liu H, Dong L, Sun H, et al. Genome sequence of the progenitor of wheat A subgenome Triticum urartu. Nature. 2018;557(7705):424-8.

5. Avni R, Lux T, Minz-Dub A, Millet E, Sela H, Distelfeld A, et al. Genome sequences of three Aegilops species of the section Sitopsis reveal phylogenetic relationships and provide resources for wheat improvement. The Plant Journal. 2022;110(1):179-92.
6. Jin X, Du H, Zhu C, Wan H, Liu F, Ruan J, et al. Haplotype-resolved genomes of wild octoploid progenitors illuminate genomic diversifications from wild relatives to cultivated strawberry. Nature Plants. 2023;9(8):1252-66.
7. Pires JC, Lim KY, Kovarík A, Matyásek R, Boyd A, Leitch AR, et al. Molecular cytogenetic analysis of recently evolved Tragopogon (Asteraceae) allopolyploids reveal a karyotype that is additive of the diploid progenitors. American Journal of Botany. 2004;91(7):1022-35.
8. Hamon P, Hamon S, Razafinarivo NJ, Guyot R, Siljak-Yakovlev S, Couturon E, et al. Coffea genome organization and evolution. Coffee in health and disease prevention: Elsevier; 2015. p. 29-37.
9. Liu S, Liu Y, Yang X, Tong C, Edwards D, Parkin IA, et al. The Brassica oleracea genome reveals the asymmetrical evolution of polyploid genomes. Nat Commun. 2014;5:3930. doi: 10.1038/ncomms4930. PubMed PMID: 24852848; PubMed Central PMCID: PMCPMC4279128.
10. Wang X, Wang H, Wang J, Sun R, Wu J, Liu S, et al. The genome of the mesopolyploid crop species Brassica rapa. Nature Genetics. 2011;43(10):1035-9. Epub 2011/08/30. doi: ng.919 [pii] 10.1038/ng.919. PubMed PMID: 21873998.
11. The Arabidopsis Genome I. Analysis of the genome sequence of the flowering plant Arabidopsis thaliana. Nature. 2000;408(6814):796-815. doi: 10.1038/35048692.
12. Burns R, Mandáková T, Gunis J, Soto-Jiménez LM, Liu C, Lysak MA, et al. Gradual evolution of allopolyploidy in Arabidopsis suecica. Nature Ecology & Evolution. 2021;5(10):1367-81. doi: 10.1038/s41559-021-01525-w.
